# Roles of activator protein-2 gamma in breast cancer: A narrative review (SANRA)

**DOI:** 10.1097/MD.0000000000030587

**Published:** 2022-09-23

**Authors:** Yifei Zhang, Asal AA Mostafa, Natthida Sriboonvorakul, Jiamiao Hu

**Affiliations:** a Engineering Research Centre of Fujian-Taiwan Special Marine Food Processing and Nutrition, Ministry of Education, Fuzhou, Fujian, China; b Food Technology Research Institute, Agriculture Research Center, Egypt; c Department of Clinical Tropical Medicine, Faculty of Tropical Medicine, Mahidol University, Bangkok, Thailand; d Faculty of Health Sciences, University of Macau, Macau, China.

**Keywords:** activator protein-2 gamma, breast cancer, transcription factor

## Abstract

Activator protein-2 gamma (AP-2γ) is a crucial transcription factor involved in breast cancer development. Abnormal expression and activity of AP-2γ have also been identified as important markers of malignancy. In the last decade, the importance of AP-2γ in breast cancer progression has been widely studied. In this review, we summarize the current knowledge on the regulatory roles of AP-2γ in breast cancer oncogenesis and progression and its potential as a diagnostic biomarker and drug target in breast cancer treatment.

## 1. Introduction

Breast cancer is the most common type of cancer among female.^[1]^ A range of modern lifestyles have been identified as risk factors contributing to the increased incidence of breast cancer, including overweight, sedentary lifestyle, processed food consumption, tobacco use, and alcohol abuse. Therefore, there is an urgent need to explore novel strategies for diagnosing and treating breast cancer. Breast cancer is a highly heterogeneous disease with various molecular features that determine its clinical response to different treatment.^[2]^ According to the gene expression profiles, breast cancer can be divided into 4 major subtypes: luminal A (estrogen receptor [ER] positive, human epidermal growth factor receptor 2 [HER2] negative, and progesterone receptor [PR] high), luminal B (ER positive, HER2 negative, and PR low), HER2 overexpression, and triple-negative/basal-like.^[3]^ Transcription factors that control gene expression, especially the signature genes for each subtype, are thought to play a critical role in the development of breast cancer and might possess great diagnostic merit.

Activator protein-2 gamma (AP-2γ) belongs to the AP-2 family of transcription factors and consists of 5 discovered members: AP-2α AP-2β, AP-2γ, AP-2δ, and AP-2ε.^[4]^ The amino acid sequences of AP-2α, AP-2β, and AP-2γ shared >75% similarity, whereas AP-2δ and AP-2ε exhibited more distinct features.^[5–7]^ Furthermore, the regulatory roles of AP-2γ transcription factors in tumor progression have been extensively investigated in many cancer types. For example, AP-2γ promotes lung tumorigenesis *via* inducing transforming growth factor-β receptor type 1–mediated p21 protein (Cdc42/Rac)-activated kinase 1 activation^[8]^ and the miR-183/miR-33a pathway.^[9]^ miR-214 contributes to melanoma tumor metastasis and progression by targeting AP-2γ,^[10]^ whereas miR-200a inhibits neuroblastoma cell proliferation by suppressing AP-2γ.^[11]^ Recently, AP-2γ was found to regulate doxorubicin resistance in osteosarcoma by forming an AP-2γ/lncRNA(LINC00922)/miRNA(miR-424-5p) feedback loop.^[12]^ AP-2γ has also been proposed as a diagnostic and prognostic marker for several types of cancer, such as adenocarcinomas, primary melanomas,^[13]^ germ cell tumors (including testicular carcinoma,^[14]^ seminomatous germ cell tumors^[15,16]^; and malignant ovarian germ cell tumors.^[17]^ Recently, the role of AP-2γ in breast cancer has been investigated extensively. For instance, the aberrant expression of AP-2γ (as well as AP-2α and AP-2β) has been reported in breast cancer.^[18]^ Particularly, AP-2γ are believed to play complex roles in the development of breast cancer.^[19]^ In this mini review, we summarize the current knowledge about the role of AP-2γ in breast cancer progression and its potential as a drug target in breast cancer treatment.

## 2. Structure of AP-2γ protein

**Figure 1.**
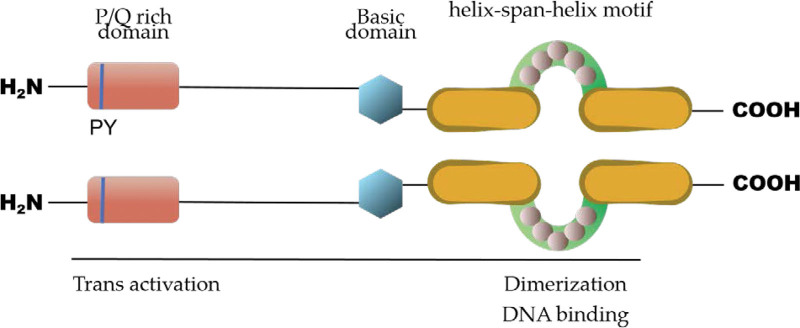
Structure of AP-2γ protein. AP-2γ protein contains a unique conserved helix-span-helix motif at the C-terminal, which mediates the dimerization of the proteins and DNA-binding together with the basic domain. Meanwhile, a proline- and glutamine-rich activation domain locates at the N-terminus of the AP-2γ protein. AP-2γ = activator protein-2 gamma.

Full-length AP-2γ protein in humans is a 48 kDa transcription factor encoded by the transcription factor-activating enhancer-binding protein 2C gene, which can bind to a range of G/C-rich elements such as DNA sequences containing a GCCNNNGGC motif.^[20]^ AP-2γ protein contains a unique conserved helix-span-helix motif at the C-terminus shared by all members of the AP-2 family, which confers the principal DNA-binding activity with an adjacent basic domain in front.^[4]^ This helix-span-helix motif also mediates homodimerization and heterodimerization between AP-2γ and other members of the AP-2 family. AP-2γ dimerization is also necessary for UBC9 (a SUMO-conjugating enzyme)–mediated sumoylation at Lysine 10 in vivo. Interestingly, this sumoylation site (at N-terminus) is far from the UBC9 interaction site (DNA-binding domain at the C-terminus), suggesting that the N-terminus of AP-2γ folds back toward the C-terminus during this posttranslational modification.^[21]^ Furthermore, the sumoylation at Lysine10 was also found to decrease the transcription activation potential of AP-2γ, highlighting the complex regulation of AP-2γ activity via multiple mechanisms, such as posttranslation modification. In addition, analysis of the AP-2γ protein revealed that its N-terminus contains a proline- and glutamine-rich activation domain. In attempts to elucidate the promoter of AP-2γ in mammary carcinoma cells, the results revealed that its promoter lacks the TATA-box and canonical binding sites for general transcription factors.^[22]^ Instead, Sp1 and Sp3 were identified to determine the transcription of AP-2γ via binding to 3 Sp1/SP3-binding sites in the CpG island in the AP-2γ promoter region^[23]^ (Fig. 1).

### 2.1. The role of AP-2γ in the development of normal breast tissues

Previous studies have suggested that AP-2**γ** is an essential transcription factor in normal breast development. The AP-2**γ** expression pattern is strongly associated with the mammary gland at developmental stages, which could be initially detected in the embryonic mammary gland and continued to increase during puberty. AP-2**γ** levels are further augmented during pregnancy and return to low levels in lactating mammary tissue. Evidence from immunohistochemistry results also demonstrated that AP-2γ was strongly expressed in the myoepithelial precursors during the budding and branching processes in fetal breast development in mice, suggesting the necessity of AP-2γ in the formation of fetal breast anlage.^[24]^ Using the AP-2γ conditional knockdown model, it was also shown that decreasing AP-2γ in mouse mammary epithelium caused a lower ratio of luminal cell population to basal cell population and aberrant growth of the mammary tree.^[25]^ Likewise, lactation deficiency caused by impaired mammary gland development was observed in transgenic mice overexpressing AP-2γ in the mammary gland. The underlying mechanism involves hyperproliferation, enhanced apoptosis, and repressed differentiation of epithelial cells in transgenic mammary glands.^[26]^ Recently, mammary stem cell–specific conditional transcription factor-activating enhancer-binding protein 2C knockout mice also demonstrated loss of AP-2γ impaired mammary ductal outgrowth due to decreased multipotency of mammary stem cells.^[27]^ Taken together, these findings highlight the indispensable role of AP-2γ in normal mammary gland development.

### 2.2. AP-2γ as a diagnostic and prognostic indicator in breast cancer

Alterations in AP-2γ expression have been determined in several clinical studies searching for novel breast diagnostic markers (Table 1). The obtained evidence showed that aberrant elevation of AP-2γ expression was observed in both luminal-like and basal-like subtypes breast cancer.^[28–33]^ Indeed, frequent amplification at regions of chromosome 20q13 where the AP-2γ gene is located is commonly found in primary breast tumors,^[34,35]^ which is believed to contribute, at least partly, to the elevated expression of AP-2γ.

**Table 1:** The association between AP-2γ expression levels and outcomes of patients with breast cancer.

AP-2γ level	Type of BC	Outcomes	Reference
↑	ERα-positive and endocrine therapy-treated patients	Disease-free survival↓ and overall survival after 10 yr↓	^[28,29]^
↑	ERα-positive with fulvestrant-treated patients	Response to fulvestrant treatment↓	^[30]^
↑(with Wwox↓)	Tamoxifen-resistant patients	Response to tamoxifen↓	^[31,32]^
↑(with CD44↓)	Patients with basal-derived breast cancer	Reaching pathologic complete response upon neoadjuvant chemotherapy↑	^[33]^

AP-2γ = activator protein-2 gamma, BC = breast cancer, ER = estrogen receptor, Wwox = WW domain containing oxidoreductase.

Moreover, AP-2γ was also found to be an important prognostic indicator for breast cancer. A number of studies have highlighted the prognostic implication of AP-2γ expression level in hormone receptor-positive breast cancer. For example, high expression of AP-2γ was reported to be associated with reduced disease-free survival^[28]^ and overall survival 10 years after diagnosis in ERα-positive and endocrine therapy-treated patients.^[29]^ In another prognostic study to search for novel markers other than PR and HER2 for the prediction of tamoxifen response, significantly increased AP-2γ and reduced WW domain containing oxidoreductase (Wwox) expressions were observed in tamoxifen-resistant tissue sections. Particularly, the nuclear AP-2γ and Wwox expression levels even showed advantages over HER2 and PR in predicting resistance in low-risk and high-risk patients.^[31]^ Considering that Wwox can inhibit AP-2γ from entering the nucleus by physically binding to AP-2γ,^[32]^ these results may indicate that AP-2γ cell distribution mediated by Wwox also plays a crucial role in determining the tamoxifen response in breast cancer. Recently, genome-wide transcriptomic analysis in 134 patients with metastatic ERα-positive breast cancer also revealed that AP-2γ levels demonstrated a strong negative correlation with the response to fulvestrant treatment.^[30]^ The evidence together strongly suggests AP-2γ expression levels possess great prognostic value for antihormone treatment. Notably, another study revealed that AP-2γ regulated epidermal growth factor receptor and receptor tyrosine kinase (RET) expressions mediate the response to vandetanib in luminal breast cancer. Since vandetanib may provide a therapeutic effect in luminal breast cancer, AP-2γ may also be used to predict the response to vandetanib treatment, together with epidermal growth factor receptor and RET.^[36]^

In addition, AP-2γ was also found to predict the outcome in patients with HER2-positive breast cancer. The AP-2γ gene signature (16-gene panel) was found to be highly predictive of distant metastasis-free survival in patients with ERα-negative, HER2-positive breast cancer.^[37]^

Interestingly, in basal-derived breast cancer, high levels of AP-2γ (together with low levels of CD44) seem to be associated with a higher chance of reaching pathologic complete response after neoadjuvant chemotherapy. Using the triple-negative cell line MDA-MB-231, it was further revealed that overexpression of AP-2γ can suppress the transcription of CD44, indicating that AP-2γ may also be an important prognostic indicator for neoadjuvant chemotherapy.^[33]^

### 2.3. The role of AP-2γ in breast cancer

Besides its crucial role in embryonic development, AP-2γ also regulates a range of cellular events in breast cancer cells. For instance, the regulatory effects of AP-2γ on the cell cycle have been well studied in breast cancer cells. Silencing of AP-2γ in MCF-7 cells caused partial G1/S arrest, the underlying mechanism of which may involve AP-2γ silencing induced active chromatin conformation at the cell cycle inhibitor p21 (CDKN1A) locus and increased CDKN1A (a cell cycle regulator) expression.^[38]^ Consistent with these results, another study conducted by Wong et al also demonstrated that MCF-7 cells with stable AP-2γ knockdown showed increased CDKN1A transcription. The AP-2γ might repress CDKN1A expression in conjunction with the oncoprotein Myc proto-oncogene, BHLH transcription factor, and lysine-specific demethylase 5B, which could form a ternary complex containing AP-2γ locating at the AP-2γ–binding site in the CDKN1A promoter and collaborate for optimal CDKN1A repression. Overexpression of these 3 proteins induced MCF-7 cells to enter the S-phase even in the presence of chemotherapy drugs with attenuated checkpoint activation, strongly suggesting that aberrant AP-2γ activity may contribute to the failure of chemotherapy in breast cancer.^[39]^ In contrast, in triple-negative MDA-MB-231 cells, overexpression of AP-2γ led to cell cycle arrest with induced CDKN1A expression,^[40]^ highlighting the AP-2γ might exert different (even contradictory) functions in cell cycle regulation in different breast cancer subtypes.

Consistently, AP-2γ was also reported to play complex roles in cell proliferation. For example, it was revealed that AP-2γ plays dual roles in HER2-positive breast cancer cell proliferation depending on its progression stages: proliferation was enhanced at early stages but reduced in advanced stages in comparison to control tumors.^[19]^ Recently, a study using a tissue-specific transgenic mouse model demonstrated overexpression of AP-2γ in mammary glands resulted in hyperproliferation of epithelial cells,^[26]^ also indicating the potential important role of AP-2γ in breast carcinoma in vivo.

In addition, as an important transcription factor in breast cancer cells, AP-2γ directly regulates the transcription of a number of genes associated with histopathological, molecular and prognostic features of breast cancer, including ERα, HER2, RET, Forkhead Box A1, GATA-Binding Protein 3 (GATA3), Myb proto-oncogene, transcription factor, e-cadherin, integrins, matrix metalloproteinases, cytokeratins, fructose-1,6-bisphosphatase, and heparanase.^[41–43]^ Interestingly, because AP-2γ recognizes GC-rich sequences, CpG islands located around the promoters of AP-2γ target genes appear to play important roles in the regulation of transcription. For example, differences in CpG island methylation between ERα-positive and ERα-negative breast cancer cells led to different accessibility of the ERα promoter for AP-2γ binding.^[44]^ Similarly, glutathione peroxidase 1, a key factor influencing the oxidative stress response in breast cancer, is mainly controlled by AP-2γ and the methylation status of the promoter. Indeed, methylation of the glutathione peroxidase 1 promoter has been identified in approximately 20% of primary breast cancers.^[45]^ RET is another good example of AP-2γ regulated proto-oncogene in breast cancer. Studies have identified 5 AP-2γ binding sites in the RET promoter which mediate AP-2γ induced RET expression.^[46]^ Interestingly, CpG island methylation of the RET promoter and decreased RET expression have been linked to poor prognosis in tumor-node-metastasis (TNM) stage II colorectal cancer patients,^[47,48]^ suggesting that epigenetic modification of CpG islands may also influence RET expression in breast cancer. Considering that promoter CpG hyper/hypomethylation is often observed in cancer, it may provide a possible explanation for why aberrance of AP-2γ mediated genes expression is common in breast cancer. Meanwhile, the interacting partners of AP-2γ in the transcription factor complex would also significantly affect the regulation of its target genes transcription. For example, the DNA repair protein Ku70 is recruited to the proximal promoter of the HER2 gene, where Ku70 physically interacts with AP-2γ to increase HER2 mRNA and protein levels in breast cancer cells.^[43]^

AP-2γ also plays an important role in the maintenance of the luminal phenotype of human breast cancer cells. Knockdown of AP-2γ in luminal breast carcinoma cells induces epithelial–mesenchymal transition with morphological and phenotypic changes as well as a decrease in luminal marker gene expression (including CD44 and E-cadherin) and an increase in basal marker gene expression (such as CD24, vimentin, and N-cadherin).^[25]^ In addition, AP-2γ represses CD44 expression in basal-derived breast cancer cells.^[33]^ Notably, AP-2γ-mediated repression of CD44 expression seems to be mediated by AP-2γ binding to the first intron of CD44, showing that AP-2γ can also regulate gene transcription in addition to binding to the traditional promoter region. Recent evidence also showed that carbonic anhydrase XII is regulated by AP-2γ by binding to its promoter region in both luminal A and luminal B breast cancers, while carbonic anhydrase XII expression is silenced in basal breast cancer *via* both CpG methylation and histone deacetylation. This finding highlights the crucial role of AP-2γ in the maintenance of the luminal breast cancer phenotype.^[49]^

### 2.4. Crosstalk between AP-2γ and ERα in breast cancer

It is widely accepted that estrogen-dependent gene regulation plays a vital role in breast tumor progression, especially in hormone-dependent breast cancer. ERα is the key receptor transducing estrogen signals into the nucleus, whose activity is also profoundly regulated by other co-factors.^[50]^ Indeed, crosstalk between AP-2γ and ERα has received significant attention in recent decades. By comparing the epigenetic chromatin structure of the ERα promoter region in ERα-negative cell lines (MDA-MB-231, MCF-10A, and MCF-7:5C) with ERα-positive cell lines (MCF-7 and T47D), we found that chromatin accessibility (measured as CpG island methylation and H3K9 deacetylation) in this region was significantly lower in ERα-negative cell lines. Furthermore, treatment with AZA/TSA in ERα-negative cell lines increased chromatin accessibility for AP-2γ and polymerase II binding at the ERα promoter, which consequently allows AP-2γ-driven ERα expression in ERα-negative cells, suggesting that AP-2γ is a crucial transcription factor for ERα gene expression in breast cancer cells.^[44]^ AP-2γ might also regulate ERα expression and activity *via* forming complexes with other proteins. AP-2γ interacts with nucleophosmin 1 to act as a transcriptional repressor of ERα in endometrial cancer cells.^[51]^ The same mechanism has not been confirmed in breast cancer cells, considering the existence of an association between endometrial and breast cancer.^[52]^ These findings also suggest that the nucleophosmin 1 and AP-2γ complex may function similarly in breast cancer.

AP-2γ has also been found to be a primary estrogen-responsive gene. Estrogen treatment significantly increased AP-2γ levels in breast tumor-derived cell lines, whereas antiestrogen 4-OH-tamoxifen or ICI 182,780 completely blocked this effect. Analysis of the 5’-UTR of the human AP-2γ gene revealed 1 consensus and 1 degenerate estrogen-responsive element, which could be recognized by ERα upon estrogen treatment.^[53]^

This mutual induction of AP-2γ and ERα gene expression may indicate a mechanism that can amplify abnormal ERα signaling after perturbation of either ERα or AP-2γ expression when oncogenesis occurs in breast tissue.

Most recently, it was demonstrated that AP-2γ can bind to ERα at binding sites in a ligand-independent manner to promote ERα transcription, showing that it might function as a pioneer factor that facilitates the efficient recruitment of ERα. Furthermore, AP-2γ binding modulates ERα-mediated long-range chromatin interactions.^[54]^ These results further highlight the involvement of AP-2γ in the regulation of ERα-mediated signaling in breast cancer cells. Collectively, the crosstalk between AP-2γ and ERα may play an important role in breast cancer progression and may be a drug target for novel breast cancer treatment.

### 2.5. AP-2γ based therapies

Although no commercial drug targeting AP-2γ has been reported, given the evidence that AP-2γ regulates a range of pathological processes associated with the breast cancer phenotype, AP-2γ is a promising drug target for breast cancer treatment. For example, AP-2γ greatly affects ERα expression levels as well as ERα signaling pathways in breast cancer and is a predominant factor in the oncogenesis and progression of breast cancer. In addition, the AP-2γ target gene RET proto-oncogene has been linked to poor prognosis in ER-positive breast cancers, and blocking the RET signaling pathway has been shown to increase sensitivity to tamoxifen in ER-positive breast cancer. Based on these findings, modulation of AP-2γ activity may provide an alternative to hormone therapy for treating hormone-dependent breast cancers. Notably, considering the complex regulatory network in breast cancer, caution should be exercised when interpreting the efficacy of interfering with AP-2γ. For example, a recent study demonstrated that decreasing AP-2γ levels in breast cancer cells resulted in high fucosyltransferase 8 expression, which significantly increased the migration ability and chemotherapy susceptibility of breast cancer cells, while AP-2γ overexpression suppressed in vivo tumor growth.^[55]^

## 3. Discussion

AP-2γ was initially identified as an important regulator of embryonic development, including in the mammary gland, and studies have also revealed its involvement in breast cancer. Several clinical studies have proposed that AP-2γ is a good diagnostic and prognostic biomarker for breast cancer patients, especially for hormone therapy and neoadjuvant chemotherapy.

Although the precise function of AP-2γ in breast cancer progression remains unclear, efforts have been made to understand its regulatory roles. Thus far, it has been clearly demonstrated that as a key transcription factor, AP-2γ may regulate the transcription of genes that fundamentally influence cellular events in breast tumor progression. However, further investigation is needed to fully understand the gene expression profiles regulated by AP-2γ as well as their significance in breast cancer.

Furthermore, recent findings also imply a possible link between AP-2γ and ERα-regulated signaling pathways, which further highlights the crucial role of AP-2γ in the oncogenesis and progression of breast cancer, especially in hormone-dependent breast cancer.

In conclusion, further investigation is needed for a more comprehensive understanding of the mechanism underlying the regulation of AP-2γ in breast cancer, which would present AP-2γ as a potential therapeutic target for breast cancer treatment.

## Author contributions

Jiamiao Hu and Yifei Zhang conceived the study and wrote the manuscript. These figures were prepared by Yifei Zhang. Mostafa Asal AA proofread the manuscript. Jiamiao Hu and Natthida Sriboonvorakul coordinated and supervised all efforts. All authors have discussed, verified, and approved the final version of the manuscript.

## References

[1] Torre LA Siegel RL Ward EM Jemal A. Global cancer incidence and mortality rates and trends--an update. Cancer Epidem Biomark Prev. 2016;25:16–27. 10.1158/1055-9965.EPI-15-0578 26667886

[2] Han B Bhowmick N Qu Y Chung S Giuliano AE Cui X. FOXC1: an emerging marker and therapeutic target for cancer. Oncogene. 2017;36:3957–63. 28288141 10.1038/onc.2017.48 PMC5652000

[3] Cancer Genome AN. Comprehensive molecular portraits of human breast tumours. Nature. 2012;490:61–70. 23000897 10.1038/nature11412 PMC3465532

[4] Eckert D Buhl S Weber S Jager R Schorle H. The AP-2 family of transcription factors. Genome Biol. 2005;6:246. 16420676 10.1186/gb-2005-6-13-246 PMC1414101

[5] Zhao F Satoda M Licht JD Hayashizaki Y Gelb BD. Cloning and characterization of a novel mouse AP-2 transcription factor, AP-2delta, with unique DNA binding and transactivation properties. J Biol Chem. 2001;276:40755–60. 11522791 10.1074/jbc.M106284200

[6] Tummala R Romano R-A Fuchs E Sinha S. Molecular cloning and characterization of AP-2ε, a fifth member of the AP-2 family. Gene. 2003;321:93–102. 14636996 10.1016/s0378-1119(03)00840-0

[7] Wang HV Vaupel K Buettner R Bosserhoff AK Moser M. Identification and embryonic expression of a new AP-2 transcription factor, AP-2 epsilon. Dev Dynam. 2004;231:128–35. 10.1002/dvdy.20119 15305293

[8] Kim W Kim E Lee S. TFAP2C-mediated upregulation of TGFBR1 promotes lung tumorigenesis and epithelial-mesenchymal transition. Exp Mol Med. 2016;48:e273e273 e273. 27885255 10.1038/emm.2016.125 PMC5133372

[9] Kang J Kim W Lee S. TFAP2C promotes lung tumorigenesis and aggressiveness through miR-183- and miR-33a-mediated cell cycle regulation. Oncogene. 2017;36:1585–96. 27593936 10.1038/onc.2016.328

[10] Penna E Orso F Cimino D. microRNA-214 contributes to melanoma tumour progression through suppression of TFAP2C. EMBO J. 2011;30:1990–2007. 21468029 10.1038/emboj.2011.102 PMC3098476

[11] Gao SL Wang LZ Liu HY Liu DL Xie LM Zhang ZW. miR-200a inhibits tumor proliferation by targeting AP-2gamma in neuroblastoma cells. Asian Pac J Cancer Prev. 2014;15:4671–6. 24969902 10.7314/apjcp.2014.15.11.4671

[12] Gu Z Zhou Y Cao C Wang X Ye Z. TFAP2C-mediated LINC00922 signaling underpins doxorubicin-resistant osteosarcoma. Biomed Pharm. 2020;129:110363. 10.1016/j.biopha.2020.110363 32563982

[13] Osella-Abate S Novelli M Quaglino P. Expression of AP-2alpha, AP-2gamma and ESDN in primary melanomas: correlation with histopathological features and potential prognostic value. J Dermatol Sci. 2012;68:202–4. 23036739 10.1016/j.jdermsci.2012.09.008

[14] Hoei-Hansen CE Nielsen JE Almstrup K. Transcription factor AP-2gamma is a developmentally regulated marker of testicular carcinoma in situ and germ cell tumors. Clin Cancer Res. 2004;10:8521–30. 15623634 10.1158/1078-0432.CCR-04-1285

[15] Biermann K Klingmuller D Koch A. Diagnostic value of markers M2A, OCT3/4, AP-2gamma, PLAP and c-KIT in the detection of extragonadal seminomas. Histopathology. 2006;49:290–7. 16918976 10.1111/j.1365-2559.2006.02496.x

[16] Pauls K Jager R Weber S. Transcription factor AP-2gamma, a novel marker of gonocytes and seminomatous germ cell tumors. Int J Cancer. 2005;115:470–7. 15700319 10.1002/ijc.20913

[17] Salonen J Leminen A Stenman UH Butzow R Heikinheimo M Heikinheimo O. Tissue AP-2gamma and Oct-3/4, and serum CA 125 as diagnostic and prognostic markers of malignant ovarian germ cell tumors. Tumour Biol. 2008;29:50–6. 18497549 10.1159/000132571

[18] Yang Y-L Zhao L-Y. Ap-2-family-of-transcription-factors-critical-regulators-of-human-development-and-cancer. J Cancer Treatment Diagnosis. 2021;5:1–4.

[19] Jäger R Friedrichs N Heim I Büttner R Schorle HJB. Dual role of AP-2gamma in ErbB-2-induced mammary tumorigenesis. Breast Cancer Res Treat. 2005;90:273–80. 15830141 10.1007/s10549-004-4815-x

[20] McPherson LA Baichwal VR Weigel RJ. Identification of ERF-1 as a member of the AP2 transcription factor family. Proc Natl Acad Sci USA. 1997;94:4342–7. 9113991 10.1073/pnas.94.9.4342 PMC20724

[21] Eloranta JJ Hurst HC. Transcription factor AP-2 interacts with the SUMO-conjugating enzyme UBC9 and is sumolated in vivo. . J Biol Chem. 2002;277:30798–804. 12072434 10.1074/jbc.M202780200

[22] Li M Wang Y Yu Y. The human transcription factor activation protein-2 gamma (AP-2gamma): gene structure, promoter, and expression in mammary carcinoma cell lines. Gene. 2002;301:43–51. 12490322 10.1016/s0378-1119(02)01057-0

[23] Hasleton MD Ibbitt JC Hurst HC. Characterization of the human activator protein-2gamma (AP-2gamma) gene: control of expression by Sp1/Sp3 in breast tumour cells. Biochem J. 2003;373(Pt 3):925–32. 12733991 10.1042/BJ20030388 PMC1223543

[24] Friedrichs N Steiner S Buettner R Knoepfle G. Immunohistochemical expression patterns of AP2alpha and AP2gamma in the developing fetal human breast. Histopathology. 2007;51:814–23. 18042070 10.1111/j.1365-2559.2007.02887.x

[25] Cyr AR Kulak MV Park JM. TFAP2C governs the luminal epithelial phenotype in mammary development and carcinogenesis. Oncogene. 2015;34:436–44. 24469049 10.1038/onc.2013.569 PMC4112181

[26] Jager R Werling U Rimpf S Jacob A Schorle H. Transcription factor AP-2gamma stimulates proliferation and apoptosis and impairs differentiation in a transgenic model. Mol Cancer Res. 2003;1:921–9. 14573793

[27] Gu VW Cho E Thompson DT Cassady VC Weigel RJ. AP-2g is required for maintenance of multipotent mammary stem cells. Stem Cell Rep. 2020;16:106–19. 10.1016/j.stemcr.2020.12.002 PMC7897584 33382976

[28] Zhao C Yasui K Lee CJ. Elevated expression levels of NCOA3, TOP1, and TFAP2C in breast tumors as predictors of poor prognosis. Cancer. 2003;98:18–23. 12833450 10.1002/cncr.11482

[29] Perkins SM Bales C Vladislav T. TFAP2C expression in breast cancer: correlation with overall survival beyond 10 years of initial diagnosis. Breast Cancer Res Treat. 2015;152:519–31. 26160249 10.1007/s10549-015-3492-2

[30] Jeselsohn R Barry WT Migliaccio I. TransCONFIRM: identification of a genetic signature of response to fulvestrant in advanced hormone receptor-positive breast cancer. Clin Cancer Res. 2016;22:5755–64. 27185372 10.1158/1078-0432.CCR-16-0148 PMC5124409

[31] Guler G Iliopoulos D Guler N Himmetoglu C Hayran M Huebner K. Wwox and Ap2gamma expression levels predict tamoxifen response. Clin Cancer Res. 2007;13:6115–21. 17947476 10.1158/1078-0432.CCR-07-1282

[32] Aqeilan R Palamarchuk A Weigel R Herrero J Pekarsky Y Croce C. Physical and functional interactions between the Wwox tumor suppressor protein and the AP-2gamma transcription factor. Cancer Res. 2004;64:8256–61. 15548692 10.1158/0008-5472.CAN-04-2055

[33] Spanheimer PM Askeland RW Kulak MV Wu T Weigel RJ. High TFAP2C/low CD44 expression is associated with an increased rate of pathologic complete response following neoadjuvant chemotherapy in breast cancer. J Surg Res. 2013;184:519–25. 23764310 10.1016/j.jss.2013.04.042 PMC3820425

[34] Kallioniemi A Kallioniemi OP Piper J. Detection and mapping of amplified DNA sequences in breast cancer by comparative genomic hybridization. Proc Natl Acad Sci USA. 1994;91:2156–60. 8134364 10.1073/pnas.91.6.2156 PMC43329

[35] Tanner MM Tirkkonen M Kallioniemi A. Amplification of chromosomal region 20q13 in invasive breast cancer: prognostic implications. Clin Cancer Res. 1995;1:1455–61. 9815944

[36] De Andrade JP Park JM Gu VW. EGFR is regulated by TFAP2C in luminal breast cancer and is a target for vandetanib. Mol Cancer Ther. 2016;15:503–11. 26832794 10.1158/1535-7163.MCT-15-0548-T PMC4783288

[37] Wu VT Kiriazov B Koch KE. A TFAP2C gene signature is predictive of outcome in HER2-positive breast cancer. Mol Cancer Res. 2020;18:46–56. 31619506 10.1158/1541-7786.MCR-19-0359 PMC6942205

[38] Williams CM Scibetta AG Friedrich JK. AP-2gamma promotes proliferation in breast tumour cells by direct repression of the CDKN1A gene. EMBO J. 2009;28:3591–601. 19798054 10.1038/emboj.2009.290 PMC2782101

[39] Wong PP Miranda F Chan KV Berlato C Hurst HC Scibetta AG. Histone demethylase KDM5B collaborates with TFAP2C and Myc to repress the cell cycle inhibitor p21(cip) (CDKN1A). Mol Cell Biol. 2012;32:1633–44. 22371483 10.1128/MCB.06373-11 PMC3347242

[40] Li H Goswami PC Domann FE. AP-2gamma induces p21 expression, arrests cell cycle, and inhibits the tumor growth of human carcinoma cells. Neoplasia. 2006;8:568–77. 16867219 10.1593/neo.06367 PMC1601932

[41] Ailan H Xiangwen X Daolong R. Identification of target genes of transcription factor activator protein 2 gamma in breast cancer cells. BMC Cancer. 2009;9:279. 19671168 10.1186/1471-2407-9-279 PMC3224728

[42] Woodfield GW Chen Y Bair TB Domann FE Weigel RJ. Identification of primary gene targets of TFAP2C in hormone responsive breast carcinoma cells. Genes Chromosomes Cancer. 2010;49:948–62. 20629094 10.1002/gcc.20807 PMC2928401

[43] Nolens G Pignon J-C Koopmansch B. Ku proteins interact with activator protein-2 transcription factors and contribute to ERBB2 overexpression in breast cancer cell lines. Breast Cancer Res. 2009;11:R83. 19906305 10.1186/bcr2450 PMC2815545

[44] Woodfield GW Hitchler MJ Chen Y Domann FE Weigel RJ. Interaction of TFAP2C with the estrogen receptor-alpha promoter is controlled by chromatin structure. Clin Cancer Res. 2009;15:3672–9. 19458056 10.1158/1078-0432.CCR-08-2343 PMC2776721

[45] Kulak MV Cyr AR Woodfield GW. Transcriptional regulation of the GPX1 gene by TFAP2C and aberrant CpG methylation in human breast cancer. Oncogene. 2013;32:4043–51. 22964634 10.1038/onc.2012.400 PMC3522755

[46] Spanheimer PM Woodfield GW Cyr AR. Expression of the RET proto-oncogene is regulated by TFAP2C in breast cancer independent of the estrogen receptor. Ann Surg Oncol. 2013;20:2204–12. 22878616 10.1245/s10434-012-2570-5 PMC3697477

[47] Chan TA Glockner S Yi JM. Convergence of mutation and epigenetic alterations identifies common genes in cancer that predict for poor prognosis. PLoS Med. 2008;5:e114. 18507500 10.1371/journal.pmed.0050114 PMC2429944

[48] Draht MX Smits KM Tournier B. Promoter CpG island methylation of RET predicts poor prognosis in stage II colorectal cancer patients. Mol Oncol. 2014;8:679–88. 24560444 10.1016/j.molonc.2014.01.011 PMC5528631

[49] Franke CM Gu VW Grimm BG. TFAP2C regulates carbonic anhydrase XII in human breast cancer. Oncogene. 2020;39:1290–301. 31636386 10.1038/s41388-019-1062-5

[50] Bernardo GM Keri RA. FOXA1: a transcription factor with parallel functions in development and cancer. Biosci Rep. 2012;32:113–30. 22115363 10.1042/BSR20110046 PMC7025859

[51] Lin CY Chao A Wang TH. Nucleophosmin/B23 is a negative regulator of estrogen receptor alpha expression via AP2gamma in endometrial cancer cells. Oncotarget. 2016;7:60038–52. 27527851 10.18632/oncotarget.11048 PMC5312367

[52] Gehrig PA Bae-Jump VL Boggess JF Groben PA Fowler WC Jr Van Le L. Association between uterine serous carcinoma and breast cancer. Gynecol Oncol. 2004;94:208–11. 15262144 10.1016/j.ygyno.2004.04.009

[53] Orso F Cottone E Hasleton MD. Activator protein-2gamma (AP-2gamma) expression is specifically induced by oestrogens through binding of the oestrogen receptor to a canonical element within the 5’-untranslated region. Biochem J. 2004;377:429–38. 14565844 10.1042/BJ20031133 PMC1223884

[54] Tan SK Lin ZH Chang CW. AP-2gamma regulates oestrogen receptor-mediated long-range chromatin interaction and gene transcription. EMBO J. 2011;30:2569–81. 21572391 10.1038/emboj.2011.151 PMC3155293

[55] Ma M Guo D Tan Z Du J Guan F Li X. Fucosyltransferase 8 regulation and breast cancer suppression by transcription factor activator protein 2γ. Cancer Sci. 2021;112:3190–204. 34036684 10.1111/cas.14987 PMC8353918

